# A novel fast method for identifying the origin of Maojian using NIR spectroscopy with deep learning algorithms

**DOI:** 10.1038/s41598-022-25671-8

**Published:** 2022-12-10

**Authors:** Chenjie Chang, Zongyuan Li, Hongyi Li, Zhuoya Hou, Enguang Zuo, Deyi Zhao, Xiaoyi Lv, Furu Zhong, Cheng Chen, Feng Tian

**Affiliations:** 1 College of Software, Xinjiang University, Urumqi, 830046 China; 2 College of Information Science and Engineering, Xinjiang University, Urumqi, 830046 China; 3 Guangzhou Panyu Polytechnic, Guangzhou, 511483 Guangdong China; 4 National Institute of Metrology, China, Peking, 100029 China; 5 School of Physics and Electronic Science, Zunyi Normal College, Zunyi, Guizhou 563006 China; 6 Xinjiang Cloud Computing Application Laboratory, Karamay, 834099 China

**Keywords:** Applied optics, Optical physics

## Abstract

Maojian is one of China’s traditional famous teas. There are many Maojian-producing areas in China. Because of different producing areas and production processes, different Maojian have different market prices. Many merchants will mix Maojian in different regions for profit, seriously disrupting the healthy tea market. Due to the similar appearance of Maojian produced in different regions, it is impossible to make a quick and objective distinction. It often requires experienced experts to identify them through multiple steps. Therefore, it is of great significance to develop a rapid and accurate method to identify different regions of Maojian to promote the standardization of the Maojian market and the development of detection technology. In this study, we propose a new method based on Near infra-red (NIR) with deep learning algorithms to distinguish different origins of Maojian. In this experiment, the NIR spectral data of Maojian from different origins are combined with the back propagation neural network (BPNN), improved AlexNet, and improved RepSet models for classification. Among them, improved RepSet has the highest accuracy of 99.30%, which is 8.67% and 0.70% higher than BPNN and improved AlexNet, respectively. The overall results show that it is feasible to use NIR and deep learning methods to quickly and accurately identify Maojian from different origins and prove an effective alternative method to discriminate different origins of Maojian.

## Introduction

Maojian is a variety of green tea rich in protein, amino acids, tea polyphenols, and other nutrients. Significantly, the selenium content is higher than other green tea, so it is widely loved by people^1,2^. Maojian produced in different regions are generally named after local names, such as Xinyang Maojian, Huangshan Maojian, and Duyun Maojian. The contents of protein, tea polyphenols, and amino acids in Maojian vary from region to region^3^. Because of different raw materials, frying processes, and quality controls, the market price and market share of different types of Maojian are also different. Sellers often mix different kinds of Maojian to make profits. This not only damaged the reputation of tea brands, violated the rights of consumers, but also seriously affected the market order.

Traditional Maojian's origin identification is mainly based on sensory evaluation, such as judging from the shape, color, aroma, and taste of Maojian. The assessment concept is vague. Subjective factors have a significant impact, and the identification efficiency is low^4^. Even tea farmers with rich experience have difficulty accurately identifying Maojian from different origins^5^. Therefore, a convenient, rapid, nondestructive, and accurate method for the identification of Maojian in different regions is needed.

Yun et al. used the headspace volatilization method and GS/MS to analyze black tea samples collected from ten geographical sources and identified 48 volatile mixtures. After that, k-nearest neighbor (k-NN) and random forest (RF) models were used to analyze the full spectrum data and 22 tea compounds, and good recognition rates were obtained^6^. Headspace GC/MS is an indirect analysis method used to determine the content of these components in the original sample through the gas components above the sample matrix. Headspace GC/MS can only detect volatile components with less than optimal parallel precision. He et al. developed a pattern recognition method to identify seasonal changes in green tea based on UPLC-QTOF/MS and chemometrics^7^. QTOF can provide high-resolution spectrograms. QTOF is fast and suitable for the analysis of large molecular weight complex samples in life sciences. Still, its cost is high, and it needs careful maintenance. Surface-Enhanced Raman Scattering (SERS) is mainly used for the qualitative and quantitative detection of tea surface contaminants and for predicting the content of certain substances in tea^8,9^. Muhammad Zaeref et al. used SERS to predict caffeine content in tea^10^. SERS data are cumbersome to prepare and have low stability. Ana Palacios-Morillo et al. applied several pattern recognition methods, such as linear discriminant analysis (LDA), support vector machines (SVM), and artificial neural networks (ANN), using UV–visible spectral data as discriminant variables to distinguish the most common tea varieties^11^. Zhang et al. used data fusion of UV–visible spectroscopy, synchronous fluorescence, NIR spectroscopy, and chemometric analysis to classify tea types. The highest classification accuracy was 97.30% using NIR spectroscopy and QDA methods^12^.

NIR spectroscopy technology is a fast and economical analysis technology. It can perform nondestructive testing without complex processing of samples and can also complete the detection of different chemical indicators^13,14^. NIR has been recognized by relevant industries for its unique advantages and is widely used in agriculture, food, ecological environment, biomedicine, and other fields^15^. As a simple and accurate detection technology, NIR is becoming more and more mature in the field of tea identification and evaluation. Wang et al. used NIR to establish an authenticity recognition model for West Lake Longjing tea and common flat tea of different years and storage periods, obtaining a 100% correct recognition rate^16^. Ren et al. used NIR and chemometrics to distinguish the origin of black tea^17^. Wang et al. used pocket-sized NIR to qualitatively and quantitatively evaluate black tea, green tea, yellow tea, and oolong tea from different countries^18^. For Pu-erh tea, Wang et al. analyzed the water-soluble metabolites of Icelandic Pu-erh tea and tea from other places based on NIR, high-resolution metabolomics, and partial least squares discriminant analysis (PLS-DA) and identified 19 characteristic compounds that can distinguish the types of Pu-erh tea, providing guidance for the identification of Pu-erh tea and helping to establish a healthy tea market^19^.

Machine learning is a mature modeling technology that allows relatively accurate models to be built by processing batch data^20^. Many examples of NIR combined with machine learning for measurement and identification have emerged in the tea field in recent years. Victor Gustavo Kelis Cardoso used NIR with SVM for data modeling, aiming to distinguish four kinds of commercial green tea mixtures, with an optimal accuracy of 93%^21^. Shih Lun Liu et al. used the discrimination model combining NIR and PLS to identify the varieties, places of origin, and seasons of tea samples. The correct recognition rates of tea samples of different varieties, places of origin, and seasons were 96.3%, 94.1%, and 99.2%, respectively^22^. Deep learning is developed from machine learning. With the increase in data scale, deep learning can learn more effective data^23^. Yang et al. combined NIR with deep learning to propose brand-new convolutional neural networks (CNN): TeaNet, TeaResnet, and TeaMobilenet to classify tea according to its quality and compared them with traditional machine learning algorithms, achieving 100% accuracy^24^. In terms of Maojian detection and classification, there is little research on applying deep learning algorithms to classify a wide range of different geographical Maojian^25^. Wang et al. discriminated the origin of Xinyang Maojian based on NIR and used statistical analysis to select the wavelength, after which the characteristic wavelengths were selected using principal component analysis (PCA) and genetic algorithm (GA), respectively, followed by PLS to predict the origin of Maojian. The results showed that GA has the highest accuracy of 97.47% for the model established by the characteristic wavelengths^26^. However, Wang et al. sampled geographically confined within Xinyang (Henan, China) and with a sample size of only 79 cases, and the GA model is prone to premature convergence when the sample size is small, making it challenging to obtain the optimal solution in some cases of high-dimensional function optimization^27^. Therefore, in this study, we will use a larger sample size to improve the model’s generalization ability, use a network structure with higher performance to avoid the problem of local optimization, and further investigate the differentiation of different geographical Maojian with larger geographical spans.

**Figure 1:**
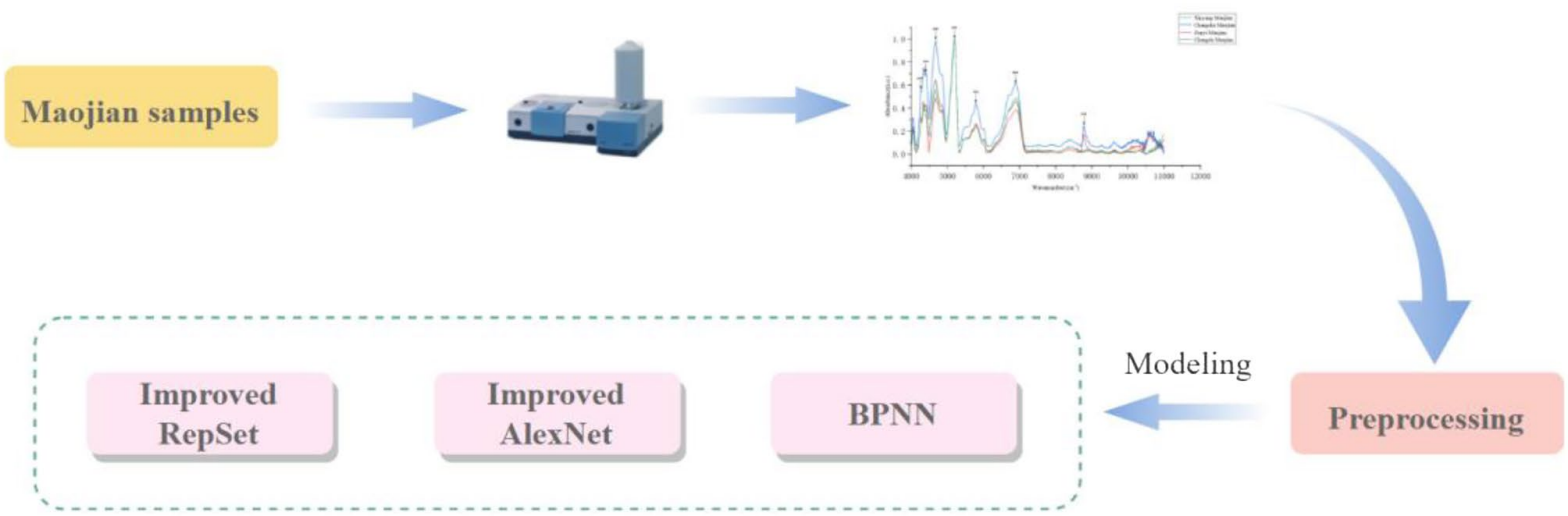
The overall work flow chart.

In this study, we establish a classification model of Maojian origin based on NIR and deep learning algorithms. BPNN, improved RepSet, and improved AlexNet are the established classification models. To improve the discriminative ability and generalization ability of the model, samples were collected from Chengdu (Sichuan, China), Zunyi (Guizhou, China), Xinyang (Henan, China), and Changsha (Hunan, China), followed by using NIR measurement samples. One hundred sample data were collected in each region, with a total of 400 sample data. The overall workflow is shown in Fig. 1. We compared the effects of different classifiers. The improved RepSet model worked the best, with an accuracy of 99.30%, which is 8.67% and 0.70% higher than BPNN and improved AlexNet, respectively. The experimental results show that the structure of the combination of the RepSet permutation invariant layer and the standard fully connected layer is more accurate in Maojian origin differentiation than some classical models proposed earlier, and it is an ideal model for identifying the origin of Maojian. Meanwhile, this study also provides a new method for classifying and identifying other types of food products.

## Experiments and methods

### Plant guidelines and sample preparation

We purchased Maojian samples from local Maojian processing enterprises in Chengdu (Sichuan, China), Zunyi (Guizhou, China), Xinyang (Henan, China), and Changsha (Hunan, China), and purchased 500 g Maojian from each production area. In the industry, because the composition of buds and leaves would involve the division of Maojian quality, to control for variables, the bud and leaf composition of the samples used in this study were all one bud and one leaf^28^. All studies for the use of plants complied with the national regulations. The prepared four types of samples were stored in a dry and airtight atmosphere at room temperature for one week, then put into a grinder to grind the four types of samples thoroughly for five minutes, and then filtered through a 200 mesh sieve. Afterward, they were put into four prepared sealed bags labeled with the corresponding origin and sealed to prevent contamination^29^.

### Measurement of NIR spectra and preprocessing

The samples were taken in sealed bags, divided into 100 portions of each type, placed in 4 ml sample tubes, and measured with a VERTEX 70 FT-IR spectrometer (Bruker, Karlsruher, Germany). Atmospheric background data were measured using OPUS 65 software (Version 6.5.83, Bruker, Germany) before each FT-IR spectral measurement^29–31^. The selected resolution is 8 cm^−1^, the number of scans is 32, and the scanning range is 4000–11,000 cm^−1^. The spectral data dimension is 1814. CO_2_ compensation is selected as the atmospheric compensation parameter. To reduce the influence of factors such as human error, we scanned each sample three times and analyzed the average spectra for subsequent analysis. Finally, we obtained 100 cases of Maojian spectral data in each region. In addition, baseline correction was done using the rubber band method to avoid the effect of electron drift and other factors on the spectra^32^. The baseline correction point value is 64. In this paper, we randomly divide the Maojian spectral data from four different origins into the training set and test set according to the ratio of 7:3. The grouped NIR data are normalized to eliminate noise interference and improve the convergence speed. In the subsequent BPNN, improved AlexNet, and improved RepSet deep learning models, we randomly selected ten samples from each class of the training set as the validation set.

### Model indicators

Table 1 shows the confusion matrix. In this paper, precision, macro avg, and accuracy indicators are used to evaluate the model performance^33^. Their formulas are as follows, where $i$ represents the $ith$ category:

1 $${\rm Accuracy } = \frac{{\rm{TP} + {\rm TN}}}{{\rm TP} +{\rm FP} + {\rm FN} + {\rm TN}}$$

2 $${\rm Precision }= \frac{{\rm{TP}}}{{{\rm TP} + {\rm FP}}}$$

3 $${\rm Macro avg} = \frac{{\sum\limits_{i = 1}^{4} {{\rm Precision}_{i} } }}{4}$$

**Table 1:** Confusion matrix.

Predicted	Actual	
	Positive	Negative
Positive	TP	FP
Negative	FN	TN

### Sample source and post experiment preservation statement

The researchers purchased Maojian samples from local tea processing manufacturers in Changsha (Hunan, China), Chengdu (Sichuan, China), Xinyang (Henan, China) and Zunyi (Guizhou, China), and visited the tea production sites. The manufacturer's tea collection process complies with local standards and national regulations (GB/T 14456.1-2017, GB/T 14456.2-2018, GB/T 14456.3-2016), and tea sales comply with Chinese laws [Food Safety Law of the People’s Republic of China (2021 Amendment)]. The purchase of Maojian samples have been authorized. The samples after the experiment are kept in the laboratory of the School of Information Science and Engineering, Xinjiang University. According to the voucher information, the samples can be kept in the laboratory for two years from May 3, 2022. The identifier is You Xue, and his email address is 601875645@qq.com.

## Results

### Spectral analysis

**Figure 2:**
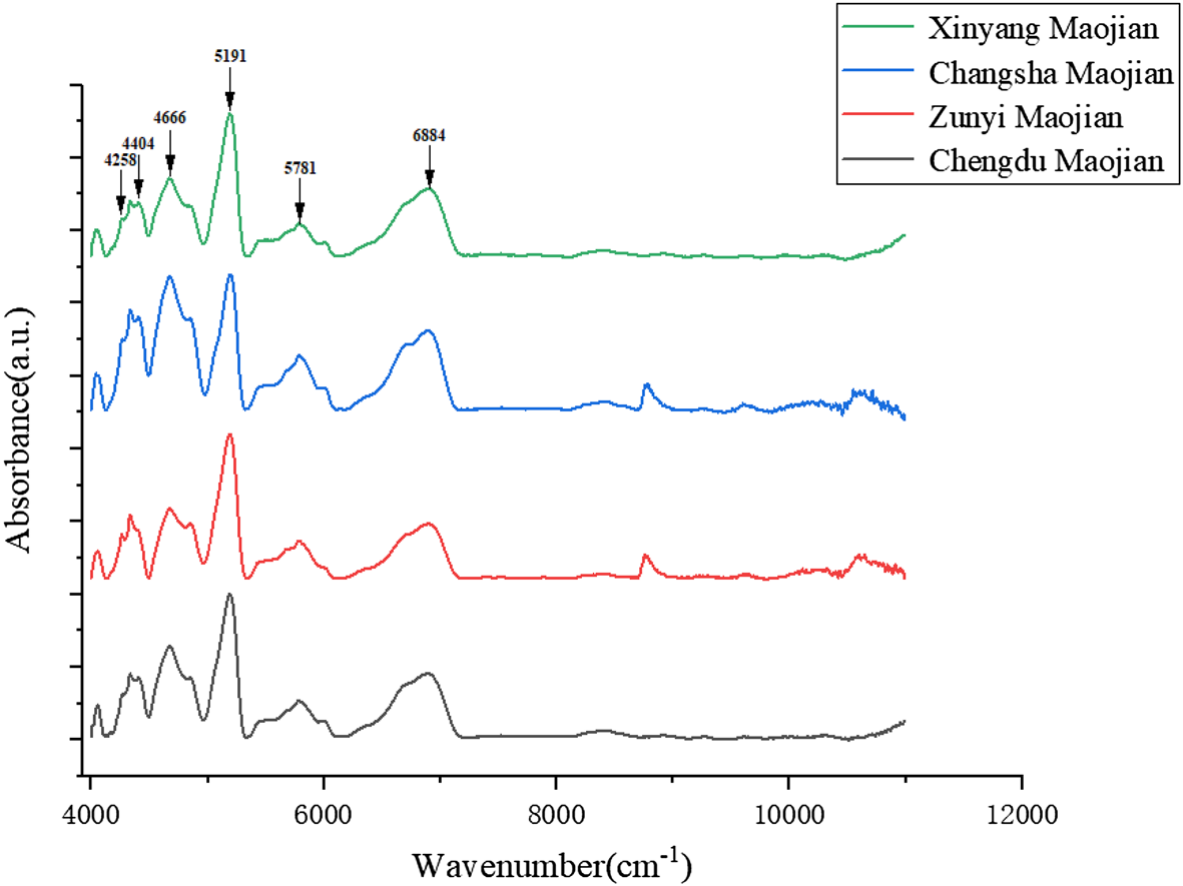
Normalized average spectral stacking line plot of Chengdu Maojian, Zunyi Maojian, Changsha Maojian, Xinyang Maojian.

**Figure 3:**
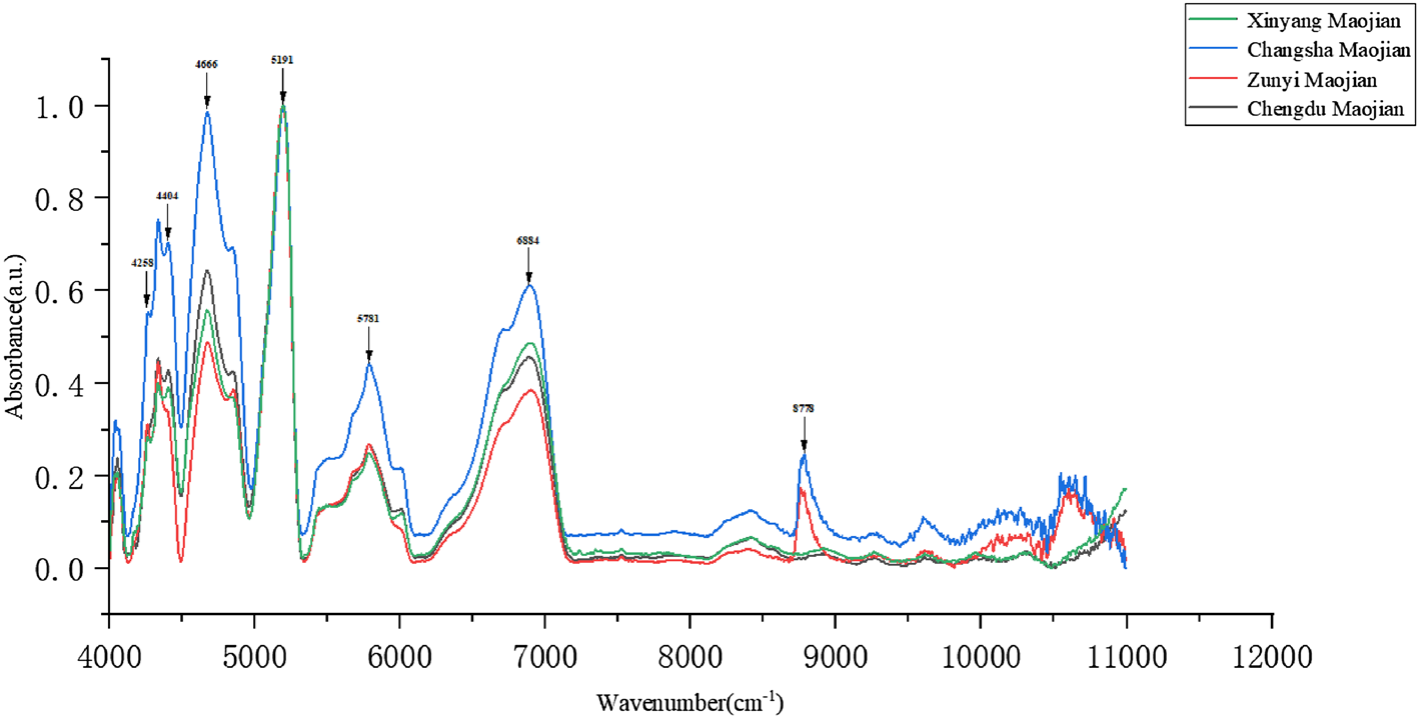
Comparison of normalized average spectral peaks of Chengdu Maojian, Zunyi Maojian, Changsha Maojian, and Xinyang Maojian.

Figure 2 shows the normalized average NIR spectral stacking line plot of Chengdu Maojian, Zunyi Maojian, Xinyang Maojian, and Changsha Maojian in the range of 4000 cm^−1^ to 11,000 cm^−1^. It can be seen from the figure that the NIR spectral peaks of Maojian from four origins are similar, with similar peaks at 4258 cm^−1^, 4404 cm^−1^, 4666 cm^−1^, 5191 cm^−1^, 5781 cm^−1^, and 6884 cm^−1^. Figure 3 is a comparison diagram of normalized average spectral peaks of four types of Maojian. The peaks at 4258 cm^−1^, 4404 cm^−1^, 4666 cm^−1^, 5781 cm^−1^ and 6884 cm^−1^ are obviously different. At 8778 cm^−1^, only Changsha Maojian and Zunyi Maojian have peaks, while Xinyang Maojian and Chengdu Maojian have no peaks. At 8778 cm^−1^, the spectral peaks of Changsha Maojian and Zunyi Maojian coincide.

According to relevant literature, the peak positions and corresponding substances are shown in Table 2^34–37^. The absorption peaks at 4258 cm^−1^ and 6884 cm^−1^ are sugar or starch^38^, 4404 cm^−1^ belongs to the absorption section of tea polysaccharides^39^, 4666 cm^−1^ and 5781 cm^−1^ belong to the absorption section of tea polyphenols^40–42^, the absorption peak at 5191 cm^−1^ belong to the absorption section of caffeine^43^, and the existence and height of 8778 cm^−1^ are due to the difference in the composition or content of certain substances in Maojian caused by local soil conditions and the production process of Maojian^44^. The substances reacted by these characteristic peaks are consistent with the substances such as tea polyphenols, caffeine, and starch contained in Maojian. From Fig. 3, it can be seen that the Maojian with high to low tea polyphenols and soluble sugar contents are Changsha Maojian, Chengdu Maojian, Xinyang Maojian, and Zunyi Maojian. The caffeine content of the four kinds of Maojian is close to each other, and the caffeine content is at a high level. The peaks of the NIR spectra represent the corresponding molecular concentration and molecular structure^29^, and the intensity of the spectral peaks of Maojian differs from region to region. Therefore, at the NIR spectral level, the biomolecular level differences between Maojian from different origins provide a solid foundation for our subsequent deep learning algorithm to distinguish Maojian from different origins.

**Table 2:** Spectral peaks with assignment of their corresponding biochemical components.

Wavenumber (cm^−1^)	Assignment
4258	C–H stretching
4404	C–H, N–N, O–H
4666	C–H stretching
5191	C=O stretching
5781	First overtone of the C–H stretching
6884	C–H stretching

### Back propagation neural network

BPNN is the most basic neural network with a three-layer structure: input layer, hidden layer, and output layer^45^. For simple feedforward neural networks, such as multi-layer perceptron (MLP), MLP only focuses on the neural network’s output without adjusting the connection weight of hidden layers^46^. BPNN uses gradient descent back-propagation to adjust the weights of network connections and uses the square of network error as the objective function to make the actual output closer to the expected output^47^. Existing studies show that artificial neural networks are suitable for modeling and classifying spectral data, and the BPNN model outperforms other data for processing NIR data^46,48,49^.

**Figure 4:**
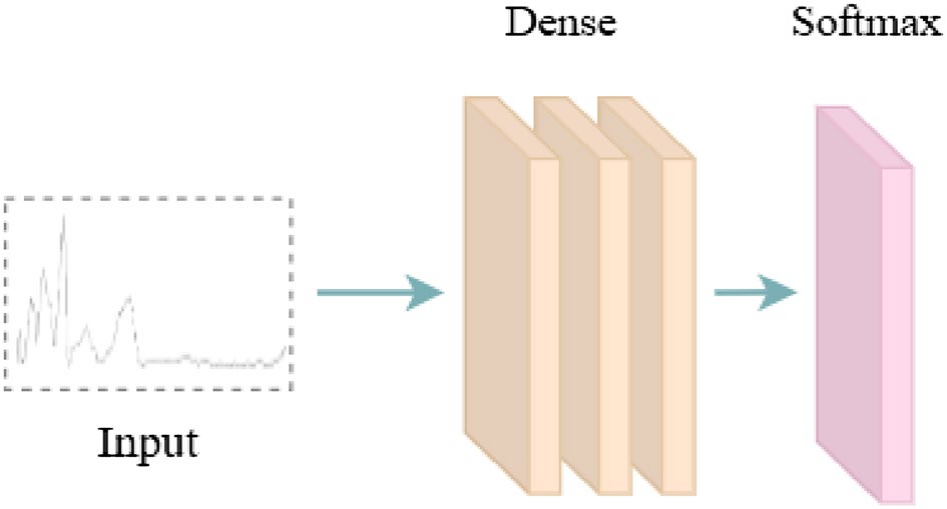
BPNN model structure.

In this paper, BPNN uses a three-layer structure to process NIR data, and the number of units in each layer is 512, 128, and 16, respectively. The network iteratively adjusts the weights of its connections to minimize the error function between the test results and the real results. The BPNN training process uses the cross-entropy loss function, and the loss function is decreased using the Adam optimization algorithm, with a learning rate of 0.001. The three-layer activation function is tanh, and the regularization term is L2. Set the batch size of training samples to 16 and the number of iterations to 80. The structure of the BPNN model is shown in Fig. 4.

The classification precision of BPNN for Changsha Maojian, Chengdu Maojian, Xinyang Maojian, and Zunyi Maojian is 100.00%, 72.00%, 95.00%, and 100.00%, respectively. Among them, the classification precision for Chengdu Maojian is low. Its macro avg is 92.00%. The recognition accuracy of BPNN for Maojian in different regions is 90.63%. The experimental results show that BPNN is an effective method to identify Maojian in different regions, but it is lower than our expectation.

### Improved AlexNet

**Figure 5:**
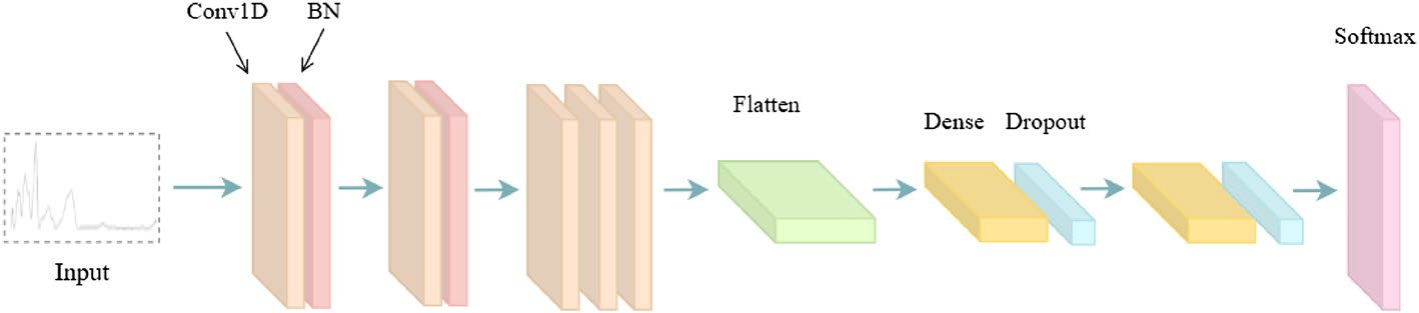
Improved AlexNet model structure.

AlexNet is a classic deep learning model. It adds the ReLU activation function behind each convolution layer, which makes the training speed of the model faster^50^. To better adapt to NIR data, this study adjusted AlexNet^31,51^. Change the two-dimensional convolution layer to the one-dimensional convolution layer. Remove all pooling layers and add batch normalization (BN) after the first three convolution layers^52^. In the adjusted AlexNet model, the activation function of each layer is activation, the optimizer is Adam, the learning rate (LR) is 0.001, and the number of iterations is 80. The improved AlexNet model is shown in Fig. 5. The experimental results show that the adjusted AlexNet model is more suitable for spectral data. The classification precision of improved AlexNet for Changsha Maojian, Chengdu Maojian, Xinyang Maojian, and Zunyi Maojian is 100.00%, 100.00%, 94.00%, and 100.00%, respectively. Among them, the classification precision of Xinyang Maojian is lower than that of other Maojian. Its macro avg is 98.00%. The classification accuracy of improved AlexNet for Maojian from different origins was 98.60%. The experimental results show that the improved AlexNet model has better classification effect on Maojian origin.

### Improved RepSet

**Figure 6:**
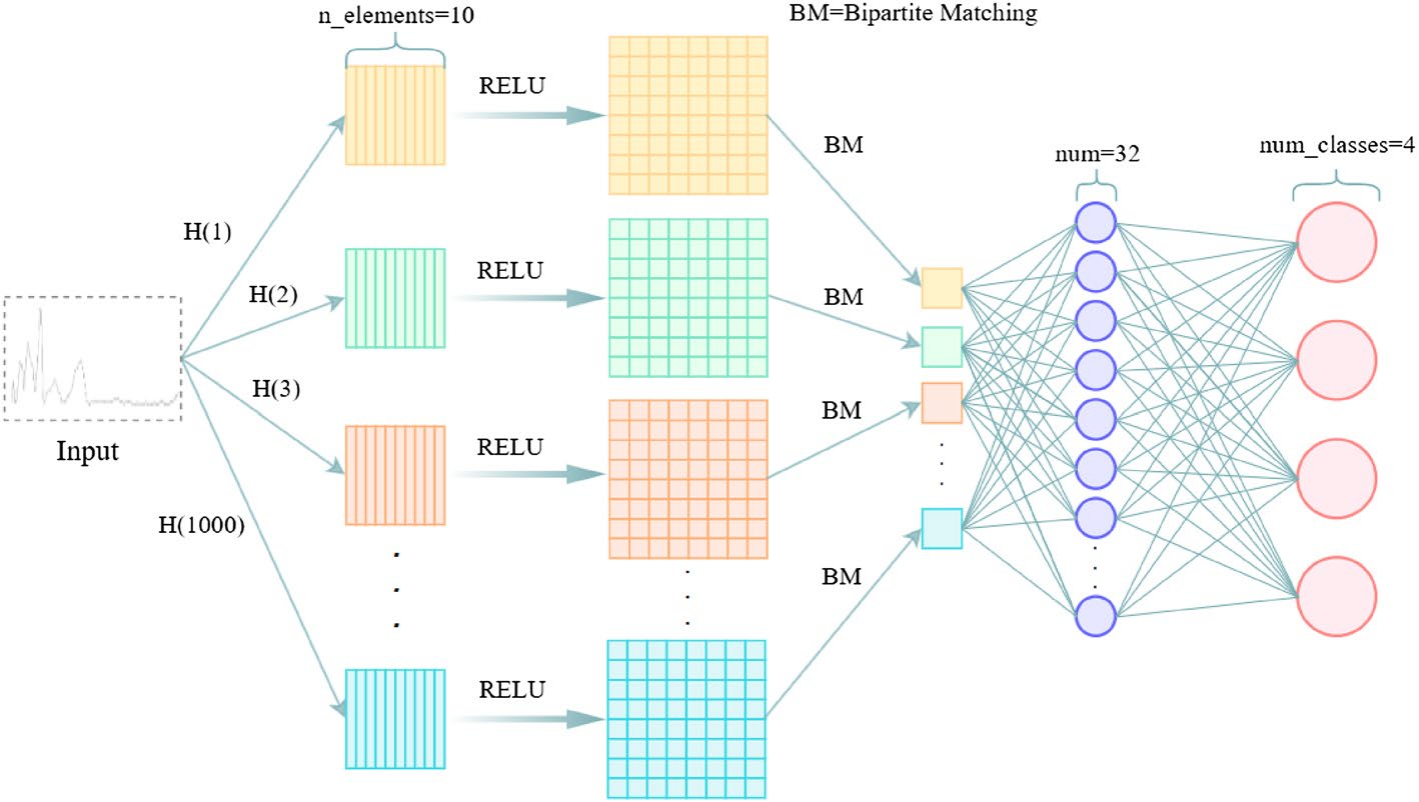
Improved RepSet model structure (this figure is the second edition. Its link is https://www.jianguoyun.com/p/DSCmu0wQmqTyCRj3vdsEIAA).

RepSet is a novel neural network architecture composed of a permutation invariant layer and standard fully connected layers. It is mainly used in the fields of computer vision and text recognition. The network architecture is used to perform learning tasks on vector sets and is capable of generating representations for unordered and variable-sized feature sets^53^. RepSet contains a certain number of hidden sets. The input set is compared with the hidden set to obtain a new matrix. The input set is compared with the new matrix using a binary matching (BM) algorithm to obtain the maximum number of matches. The maximum number of matches is fed into the fully connected layer to output classification results. To adapt to the NIR data, we adjusted the RepSet model structure. The adjusted improved RepSet model structure is shown in Fig. 6.

The dichotomous matching problem is the most studied problem in combinatorial optimization. It mainly studies the problem of no relationship between the elements of two sets themselves. For the problem that the elements of two sets are related, the related elements can be matched to get the maximum matching number. The maximum matching formula is as follows:

$$\begin{gathered} \max \sum\limits_{i = 1}^{|X|} {\sum\limits_{{{\text{j}} = 1}}^{{|{\text{Y}}|}} {x_{ij} } } f(v_{i} ,u_{j} ) \hfill \\ {\text{Subject to}}: \hfill \\ \end{gathered}$$

4 $$\sum\limits_{i = 1}^{|X|} {x_{ij} \le 1} \quad \forall {\text{j}} \in \{ 1, \ldots ,|Y|\}$$

$$\sum\limits_{j = 1}^{|Y|} {x_{ij} \le 1} \quad \forall i \in \{ 1,\ldots,|X|\}$$

$$x_{ij} \ge 0\quad \forall i \in \{ 1,\ldots,|X|\} ,\forall j \in \{ 1,\ldots,|Y|\}$$

Given the input set $X = \{ v_{1} ,v_{2} ,\ldots,v_{|X|} \}$ and the hidden set $Y = \{ u_{1} ,u_{2} ,\ldots,u_{|Y|} \}$. $|X|$ and $|Y|$ are the cardinality of $X$ and $Y$, respectively. $f(v_{i} ,u_{j} )$ is a differentiable function. If the component $i$ of $X$ is assigned to the component $j$ of $Y$, then $x_{ij} = 1$, otherwise $x_{ij} = 0$. In this experiment, $f(v_{i} ,u_{j} )$ will be defined as the inner product of $v_{i}$ and $u_{j}$, followed by the ReLU activation function. Hence, $f(v_{i} ,u_{j} ) = {\text{ReLU}}(v_{i}^{T} u_{j} )$.

**Figure 7:**
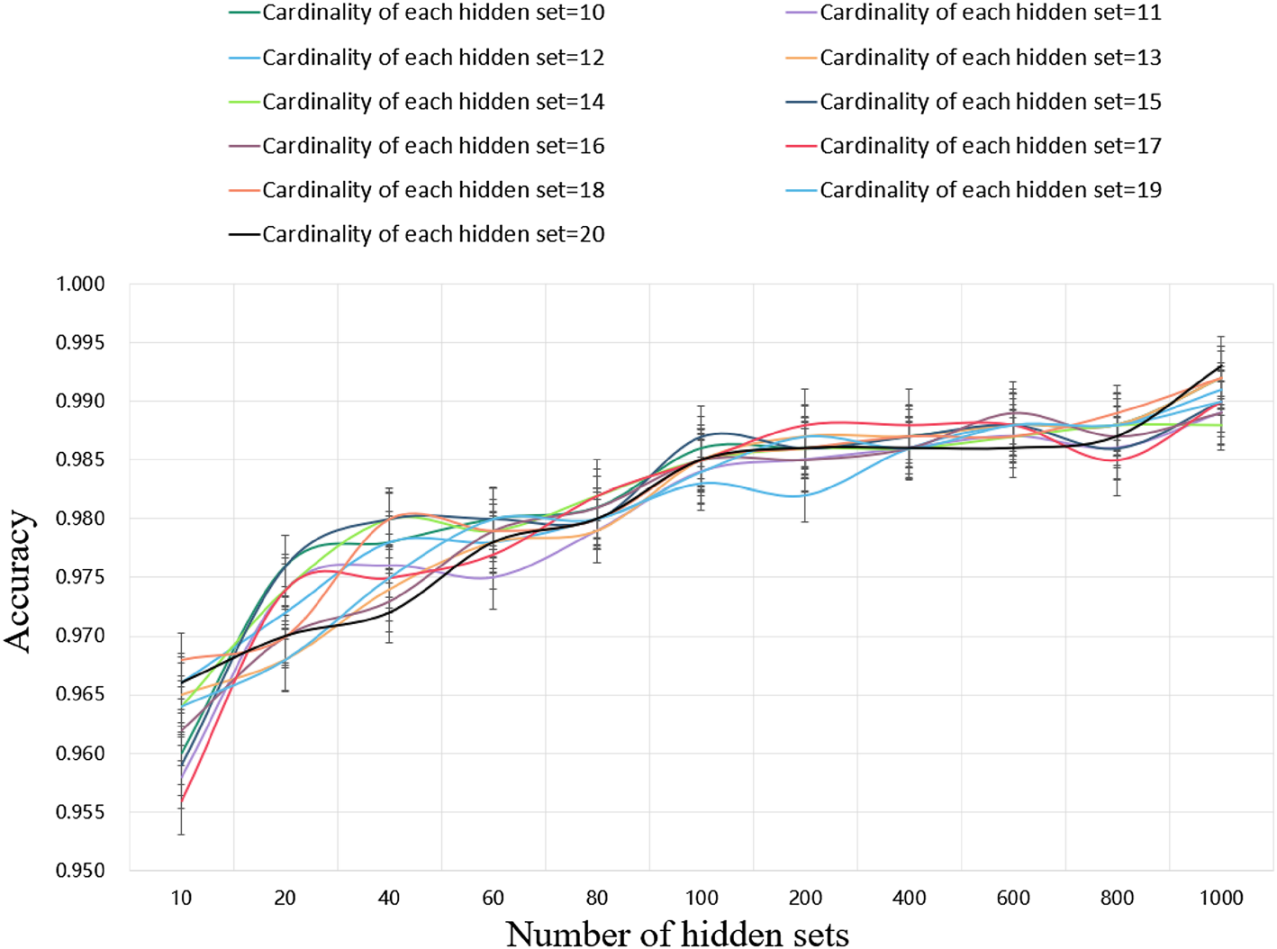
The accuracy of the improved RepSet model for the classification of four regional Maojian with different number of hidden sets and different cardinality of hidden sets.

Given the number of hidden sets, the cardinality of each hidden set, and the dimension of each vector, the hidden set is returned by the randn function, which is the standard normal distribution and trainable. The number of different hidden sets and the cardinality of each hidden set have a certain impact on the model effect. In this experiment, we studied the influence of the number of hidden sets and the cardinality of each hidden set on the accuracy of Maojian classification in four regions. Limited by the performance of the computer CPU (i5-9400f), the value range of the cardinality of hidden sets in this experiment is 10 to 20, and the value range of the number of hidden sets is 10 to 1000. Using the control variable method, the classification accuracy under different parameters is shown in Fig. 7. It can be seen from the figure that the number of hidden sets is positively correlated with the classification accuracy, but there is no obvious relationship between the cardinality of hidden sets and the accuracy. With the increase in the number of hidden sets, the accuracy increases. When the number of hidden sets is 1000, and the cardinality of hidden sets is 20, the accuracy rate is the highest.

In this experiment, we set the parameters of the improved RepSet network structure as follows: the number of iterations is 30, the learning rate is 0.001, the batch size is 20, the number of hidden sets is 1000, the cardinality of hidden sets is 20, and the number of neural units in the two fully connected layers is 32 and 4, respectively. Table 3 shows that the classification precision of improved RepSet for Changsha Maojian, Chengdu Maojian, Xinyang Maojian, and Zunyi Maojian is 100.00%, 100.00%, 99.00%, and 100.00%, respectively. Its macro avg is 99.75%. The classification accuracy of the improved RepSet in distinguishing four regions is 99.3%. The experimental results show that improved RspSet is particularly an accurate and efficient method to identify the origin of Maojian.

**Table 3:** Classification precision of different models for different geostrophic Maojian.

Model	Maojian			
	Changsha Maojian (%)	Chengdu Maojian (%)	Xinyang Maojian (%)	Zunyi Maojian (%)
BPNN	100.00	72.00	95.00	100.00
Improved AlexNet	100.00	100.00	94.00	100.00
Improved RepSet	100.00	100.00	99.00	100.00

## Discussion and conclusion

In this study, we identified Maojian from Chengdu, Zunyi, Xinyang, and Changsha through different deep learning algorithms combined with NIR spectral data. We first analyzed the spectra of Maojian in different regions and found that they had similar NIR spectra, but the intensity of the spectral peaks was different, indicating the different molecular concentrations or contents, which provided a solid basis for us to distinguish Maojian from different origins using NIR spectra and deep learning algorithms. In this paper, we used the traditional BPNN model, the improved AlexNet model adapted to NIR after adjustment, and a new improved RepSet model after adjustment. As shown in Table 4, the classification accuracy of Maojian in four regions is 90.63%, 98.60%, and 99.30%, respectively. Among them, the improved RepSet model has the best effect, 8.67% and 0.70% higher than BPNN and improved AlexNet. We discussed the number of hidden layers and the cardinality of hidden layers in the improved RepSet structure. According to the experimental results, we finally selected the number of hidden layers as 1000 and the cardinality of hidden layers as 20. The experimental results of this paper show that the proposed model realizes the efficient and accurate classification of four different origins of Maojian and overcomes the shortcoming of subjectivity in identifying different origins of Maojian. Due to the sufficient sample size, the generalization ability of the model was also improved. The use of NIR combined with deep learning algorithms in this study also provides a new approach for classifying and identifying other types of food products.

**Table 4:** Classification accuracy of different models.

Model	BPNN	Improved AlexNet	Improved RepSet
Accuracy	90.63%	99.30%	98.60%

## Data Availability

The datasets generated and analysed during the current study are not publicly available due to the nature of this research but are available from the corresponding author on reasonable request.
